# Decision tools for bacterial blight resistance gene deployment in rice-based agricultural ecosystems

**DOI:** 10.3389/fpls.2015.00305

**Published:** 2015-05-05

**Authors:** Gerbert S. Dossa, Adam Sparks, Casiana Vera Cruz, Ricardo Oliva

**Affiliations:** ^1^Plant Breeding, Genetics, and Biotechnology Division, International Rice Research Institute, Metro Manila, Philippines; ^2^Department of Phytomedicine, Leibniz Universität Hannover, Hannover, Germany; ^3^Crop and Environmental Sciences Division, International Rice Research Institute, Metro Manila, Philippines

**Keywords:** customized deployment, forward breeding, TAL effectors, R-genes, genome editing

## Abstract

Attempting to achieve long-lasting and stable resistance using uniformly deployed rice varieties is not a sustainable approach. The real situation appears to be much more complex and dynamic, one in which pathogens quickly adapt to resistant varieties. To prevent disease epidemics, deployment should be customized and this decision will require interdisciplinary actions. This perspective article aims to highlight the current progress on disease resistance deployment to control bacterial blight in rice. Although the model system rice-*Xanthomonas oryzae* pv. *oryzae* has distinctive features that underpin the need for a case-by-case analysis, strategies to integrate those elements into a unique decision tool could be easily extended to other crops.

## Why Customize the Deployment of Bacterial Blight Resistance Genes?

Very often, elite rice varieties carrying effective resistance genes are distributed across broad geographic areas to maximize their socioeconomic impact. Eventually, the resistance gene will overlap with virulent pathogen populations that persist in low frequency. Prolonged exposure will increase selected population and leads to an outbreak. In this case, deployment is perhaps the most influential event compromising durability. Preventing disease epidemics requires a deeper understanding of the biological systems and interdisciplinary approaches to interconnect the factors that account for conducive environments, locally effective genes, and pathogen dynamics. A systematic monitoring of the pathogen population, which incorporates current understanding of effector biology, emerges as a key aspect to drive pathogen-informed deployment. However, it is essential that such information is readily transferred to breeding pipelines to guarantee the right variety profiles. We also believe that geographic information systems can be used to couple disease forecasting models with in-field surveys and other on-the-ground work to map epidemics in real time and therefore be integrated into a unique decision tool.

Bacterial blight (BB), caused by *Xanthomonas oryzae* pv. *oryzae* (*Xoo*), is the most important bacterial disease of rice. At least 39 resistance genes (*Xa*) have been identified from wild and cultivated accessions (Khan et al., 2014; Zhang et al., 2014a); among which *Xa4*, *xa5*, *xa13*, and *Xa21* appear to be widely used in breeding programs across Asia (Khan et al., 2014). Disease resistance deployment to control *Xoo* emerges as a perfect case for analysis because (i) the disease is widely distributed across rice-growing regions worldwide, (ii) resistance has a strong race-specific component, and (iii) many *Xa* genes have been incorporated into released varieties. A game-changing feature of the pathosystem is that *Xoo* uses transcription activator-like (TAL) effectors to promote colonization and ensure nutrient uptake. In contrast to other rice pathosystems, *Xa* genes can be classified into distinct functional categories and only a small number appears to encode for NBS-LRR proteins (Boch et al., 2014). In this paper, we describe the key elements that need to be considered if we are to implement a strategy to customize the deployment of *Xa* genes in rice agroecosystems.

## Breeding Fast, Breeding Precisely

Conventional breeding in rice uses pedigree breeding and selection, which employs a forward breeding approach based on traits of interest. The selection of advanced pedigree lines and recombinant inbred lines requires a long process that can take 8-9 years to generate elite lines for varietal release. With the recent advances in technologies, breeding for target traits can be fast-tracked by the application of marker-assisted selection (MAS) focusing on improving tolerance to abiotic stresses—drought, submergence, salinity, and soil problems—for unfavorable environments, along with increased yield, biotic stress resistance, and improved grain quality, traits that are also required for a favorable environment. Recently, the duration for developing improved varieties through forward breeding was decreased to 5-6 years through implementing rapid generation advance and MAS techniques. Further enhancement uses marker-assisted backcrossing (MABC) retaining the good traits of the recipient parents, combined with precision in incorporating specific traits of interest into high-yielding mega-varieties with superior grain quality, thus further reducing the time of producing elite lines to 3-4 years. Recently, a further transformation in breeding has been revolutionizing precision breeding while doubling the rate of genetic gains. The availability of 3,000 sequenced rice genomes provides an unprecedented wealth of data to mine alleles for novel genes (Li et al., 2014). This is coupled with advances in high-throughput SNP genotyping across large breeding populations in various platforms (Fluidigm’s Dynamic Arrays^TM^, Douglas Scientific Array Tape^TM^, and LGC’s automated systems for running KASP^TM^ markers) to accelerate rice improvement (Thomson, 2014). Likewise, genotyping by sequencing (GBS) is currently becoming a choice for low-cost high-density genome-wide scans using multiplexed sequencing.

**FIGURE 1:**
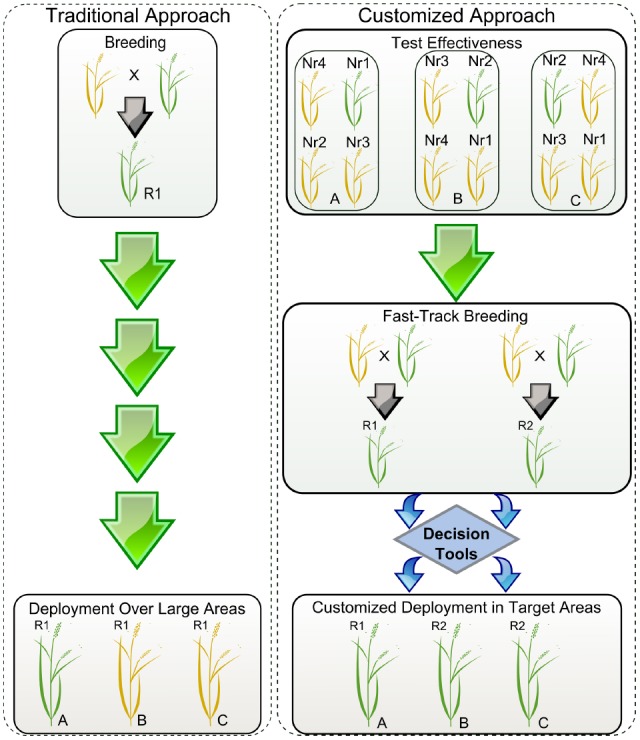
**Simplified scheme showing the deployment of resistant varieties using traditional versus customized approaches.** R1 and R2 represent resistant elite varieties carrying hypothetical genes 1 and 2. Yellow and green plants represent susceptible and resistant phenotypes, respectively. Locations A, B, and C represent cropping regions that do not share boundaries. Nr1, Nr2, Nr3, and Nr4 are near-isogenic lines (NILs) for each of the available resistance genes 1, 2, 3, and 4. During traditional deployment, variety R1 is bred and released in large areas but is effective only in particular locations. During customized deployment, the effectiveness of the resistance genes and pathogen population structures are monitored using disease hotspots, seasonal collections, and pathogenicity tests done in a confined setting. Using a decision tool, breeding programs can rapidly customize the elite varieties to be deployed in targeted locations based on variety profiles.

At IRRI, in-house genotyping services for various breeding programs contribute to fast-tracking the breeding cycle from hybridization to population advancement in 2-3 years to generate elite lines. Structurally, IRRI breeding hubs facilitate multi-environment testing with strategically selected locations in South Asia (India), Southeast Asia (Myanmar and the Philippines), and East and Southern Africa (Burundi), in partnership with other partners from both the public and private sector. Through this transformed breeding process, we envision generating various combinations of effective *Xa* genes in similar or different elite backgrounds for market segmentation. The strategy will likely promote shuffling of resistance mechanisms displayed on the field to prevent rapid pathogen adaptation to single-gene virulence, deployed in a customized manner (Figure 1). Real-time deployment of resistant varieties with various combinations of effective *Xa* genes can be customized through gene rotation or mixture in a single genetic background targeted for evolving or dynamic pathogen populations, especially in a BB-endemic rice-growing environment. In a broader context, breeding programs are allowed to prioritize genes that are effective across multiple locations, but also genes that combine different mechanisms and show low turnover rate.

## Monitoring of Effective Genes

**FIGURE 2:**
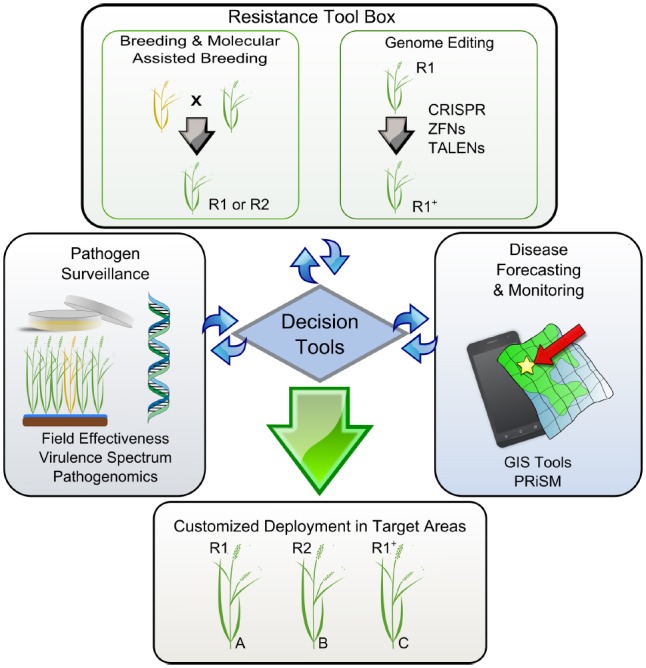
**A model representing key elements supporting the decision tools for customized deployment of resistance genes in rice.** Disease-prone areas are predicted and geo-referenced with other environmental constraints using GIS. Pathogen surveillance and the effectiveness of R-genes can be adapted to a seasonal base and used by breeding programs to timely direct breeding efforts. R1 and R2 represent resistant elite varieties carrying hypothetical genes 1 and 2. Yellow and green plants represent susceptible and resistant phenotypes, respectively. Locations A, B, and C represent cropping regions that do not share boundaries. A resistance tool kit provides adequate technologies that allow fast-tracking the response to particular needs. For instance, R1^+^ represents an elite variety with an artificially expanded spectrum of recognition that can be deployed in additional areas. All elements are gathered and interconnected through a unique platform (decision tool) for customized deployment in targeted areas.

In order to customize deployment, we need to assess the effectiveness of R-genes in specific target areas and understand the evolutionary potential of the local *Xoo* population. Seasonal monitoring, when timely executed and concerted in a cost-effective manner, could be very useful for breeders to direct breeding efforts (Figure 2). The first aspect of monitoring entails a collection of field strains to understand population genetic structure and local distribution. So far, all markers based on restriction fragment length polymorphism profiling or repeated sequences (Choi et al., 1998; Chen et al., 2012; Mishra et al., 2013) have not succeeded in describing functional groups or simply are not currently available in high-throughput platforms. However, many large-scale shotgun sequencing projects involving short-reads are underway and would potentially allow us to identify a set of SNP markers for standard use in field genotyping across different regions. Ultimately, the hope is that current advances in long-reads sequencing chemistry will resolve TAL effector sequences within complex samples, thus opening the door for more informative monitoring initiatives. The second aspect may involve the use of near-isogenic lines (NILs) carrying updated sets of *Xa* genes. Traditionally, this material has been used as a tool to identify phenotypic groups under controlled conditions (Ogawa et al., 1988). NILs are also suitable for deployment in disease-endemic areas or “hotspots” to capture low-prevalence genotypes that might escape from seasonal collection or to assess the effectiveness of *Xa* genes at a local scale (Figure 1). Ideally, both aspects could provide regional breeders with real-time decision support for small-scale interventions. Since 2013, IRRI has been deploying NILs in target areas of Asia and Africa and coordinating efforts to collect *Xoo* samples with local partners. Lessons learned from engaging rice research programs suggest that monitoring must be a participatory exercise and information exchange between national partners is more likely to occur under a common platform.

## Exploiting Effectors to Drive Deployment

Scientists defined effectors as molecular instruments that facilitate a parasitic life style (Hogenhout et al., 2009). Translational research done in a number of crops has tailored effector biology into a useful tool for disease resistance breeding (Gawehns et al., 2013; Vleeshouwers and Oliver, 2014). In oomycetes and fungal pathosystems, effectors have been used to facilitate cloning R-genes, discovering novel specificities, or avoiding unnecessary breeding effort (Vleeshouwers et al., 2011; Oliver et al., 2012; Rietman et al., 2012). Recent advances in the biochemical function of bacteria TAL effectors suggest that their activity has a major impact on virulence (Kay and Bonas, 2009). TAL effectors activate the expression of specific host susceptibility genes (*S*) in order to create a favorable environment. For instance, increasing sucrose availability or reducing copper-mediated toxicity within the xylem vessels appears to be a clear output of its virulence function during rice-*Xoo* interaction (Chen et al., 2010; Yuan et al., 2010). With the TAL-DNA recognition decoded (Boch et al., 2009; Moscou and Bogdanove, 2009), it is now possible to have a catalog of validated TAL targets in the rice genome (Noël et al., 2013). The increasing evidence that TAL repertoires can determine host specificity in a gene-for-gene fashion (Yang and White, 2004; Gu et al., 2005; Yang et al., 2006; Antony et al., 2010; Tian et al., 2014; Wang et al., 2014) highlights their potential use in translational research. For instance, *Xoo* strains carrying TAL effectors *Avrxa10*, *AvrXa23*, and *AvrXa27* are unable to colonize rice accession containing *Xa10*, *Xa23* and *Xa27*, respectively (Gu et al., 2009; Tian et al., 2014; Wang et al., 2014).

Theoretically, the allelic diversity of TAL effectors in a region can be used to help deployment interventions, but in practice we need to overcome some technical issues: (i) What is the best way to efficiently capture TAL repertoires (TALome)? Probably, a combination of TAL enrichment methods and high-throughput sequencing using long-reads will be enough to map the TALome, although cost-efficiency remains a major limitation. (ii) How do we improve current algorithms to efficiently catalog TAL targets? While many candidate *S-*genes have been validated experimentally, we expect an enhanced accuracy of prediction tools (Doyle et al., 2012; Grau et al., 2013; Pérez-Quintero et al., 2013) as more *Xoo* genomes become available and the algorithms can be trained on natural alleles of candidate targets using enlarged rice data sets (Li et al., 2014). (iii) Is it worthwhile assessing epiallelic variation of TAL effectors among field isolates? So far, no evidence suggests that *Xoo* actively uses this pathway to adapt to the selection pressure imposed by agricultural deployment. For now, it does not seem likely that a diagnostic test will be included in regular surveys because detecting gene expression in field samples can be technically challenging and economically not feasible (Gijzen et al., 2014). In summary, we cannot afford to exclude effectors-based information from a modern rice breeding and deployment program, but addressing these questions is essential for fine tuning the overall strategy. Beside effectors biology, the study of cell to cell signaling pathways or virulence regulation in response to abiotic factors are some of the emerging areas of research that will need some attention in the near future.

## Mapping Disease in Real Time

The technologies currently exist to merge all of these concepts into a working system for targeted deployment. For example, the Philippine Rice Information System (PRiSM^1^) gathers data from farmers’ fields throughout the Philippines. Trained individuals use a standardized survey portfolio based on the IRRI publication “A Survey Portfolio to Characterize Yield-Reducing Factors in Rice” (Savary and Castilla, 2009), collecting data on yield-reducing (biotic stresses) and also yield-limiting (abiotic stresses) factors, current yields, and farmers’ agronomic practices using smartphones. The data are submitted via a wireless connection (Wi-Fi or cellular) to a centralized database in near real time. These data allow us to create a profile of a local targeted environment for breeders to reference by linking with other sources of information. The profile could include the most common pathogen population structure (collected in the fields being surveyed), farmers’ market preferences, farmers’ agronomic practices, and biotic and abiotic stresses in the target area. For example, using this technology, breeders working to develop a variety for an area where flooding is common could see where BB and flooding are most likely to occur together. Then, these data could be coupled with the local *Xoo* population structure and local market preferences to develop a variety that farmers would be likely to adopt and that is tailored to that specific environment’s stresses (Figure 2).

## Broadening the Spectrum of Recognition, Only if Necessary

Genome editing techniques enable targeting specific DNA sequences and introducing a broad range of precise genetic modifications (Fichtner et al., 2014). Beside the current direction of the regulatory debate, the outcome of this technology is not a GMO product because it does not contain any foreign DNA (Waltz, 2012). While most of the available genome editing tools (ZFNs, TALENs, CRISPR-Cas9) have been successfully tested in rice (Li et al., 2012; Miao et al., 2013; Zhang et al., 2014b; Zhou et al., 2014), current progress on virulence mechanisms promoted by TAL effectors has inspired new ways to immunize crops using such an approach. For instance, resistance can be acquired by disrupting the TAL effector binding site of major *S*-gene promoters, such as members of the SWEET sucrose-efflux transporter family (Li et al., 2012). Eventually, broad-spectrum resistance can be created if several family members are targeted at the same time, thus limiting the access of the bacteria to alternative nutrient resources. Resistance can also be engineered using multiple decoy TAL-binding sites fused upstream of a single executor R-gene (Römer et al., 2009). Among all the rice R-genes that have been reported (Gu et al., 2005; Tian et al., 2014; Wang et al., 2014), few have potential applications as executors because they trigger strong localized cell-death and are induced only in the presence of the pathogen. We predict that genome editing tools will be integrated into the next-generation resistance tool kit, but might be considered only when no other alternative is available (Figure 2). For instance, elite varieties with an artificially expanded spectrum of recognition (either *R* or *S*) may become a solution in regions where management practices or pyramiding of existing *Xa* genes are no longer options. Swarna-Sub1 is a high-yielding mega-variety that shows a yield advantage in flood-prone areas of Asia (Ismail et al., 2013) but it is quite susceptible to *Xoo* infection in some of these unfavorable environments. Current attempts to precisely fast-track effective combinations of *Xa* genes into Swarna-Sub1 are under way, but the number of effective *Xa* genes available is limited and alternative strategies to increase the diversity of mechanisms are key for sustainable deployment-based management (Figure 2). Whether we are planning to exploit *R-* or *S*-genes to broaden the spectrum of resistance, it is clear that genome editing tools will be important assets for next-generation resistance breeding.

## One Tool to Rule Them All

It is only a matter of time before information and communication technologies (ICTs) lead the research revolution on the agricultural landscape. Currently, very precise information can be retrieved and/or delivered to and from farmers’ hands in real time. Platforms that incorporate crop health status under well-characterized environments are coming and will soon become tools for informed interventions. Therefore, it becomes very important that pathologists, breeders, and epidemiologists endeavor to integrate diagnostics, disease models, and breeding efforts into a unique platform for customized deployment (Figure 2). This vision does not exclude other fields of research that also contribute to increased variety adoption and are important in the rice value chain. These are exciting times, like never before, rice scientists have the possibility to adapt their breeding programs and decide which variety will be promoted next season to reduce the chance of future epidemics.

### Conflict of Interest Statement

The authors declare that the research was conducted in the absence of any commercial or financial relationships that could be construed as a potential conflict of interest.

## Footnotes

1 http://philippinericeinfo.ph
